# Potential role of nuclear PD-L1 expression in cell-surface vimentin positive circulating tumor cells as a prognostic marker in cancer patients

**DOI:** 10.1038/srep28910

**Published:** 2016-07-01

**Authors:** Arun Satelli, Izhar Singh Batth, Zachary Brownlee, Christina Rojas, Qing H. Meng, Scott Kopetz, Shulin Li

**Affiliations:** 1 Department of Pediatrics, The University of Texas MD Anderson Cancer Center, Houston, Texas, USA; 2 Department of Laboratory Medicine, The University of Texas MD Anderson Cancer Center, Houston, Texas, USA; 3 Departments of Surgical Oncology and Molecular and Cellular Oncology, The University of Texas MD Anderson Cancer Center, Houston, Texas, USA; 4 The University of Texas Graduate School of Biomedical Sciences, Houston, Texas, USA.

## Abstract

Although circulating tumor cells (CTCs) have potential as diagnostic biomarkers for cancer, determining their prognostic role in cancer patients undergoing treatment is a challenge. We evaluated the prognostic value of programmed death-ligand 1 (PD-L1) expression in CTCs in colorectal and prostate cancer patients undergoing treatment. Peripheral blood samples were collected from 62 metastatic colorectal cancer patients and 30 metastatic prostate cancer patients. CTCs were isolated from the samples using magnetic separation with the cell-surface vimentin(CSV)-specific 84-1 monoclonal antibody that detects epithelial-mesenchymal transitioned (EMT) CTCs. CTCs were enumerated and analyzed for PD-L1 expression using confocal microscopy. PD-L1 expression was detectable in CTCs and was localized in the membrane and/or cytoplasm and nucleus. CTC detection alone was not associated with poor progression-free or overall survival in colorectal cancer or prostate cancer patients, but nuclear PD-L1 (nPD-L1) expression in these patients was significantly associated with short survival durations. These results demonstrated that nPD-L1 has potential as a clinically relevant prognostic biomarker for colorectal and prostate cancer. Our data thus suggested that use of CTC-based models of cancer for risk assessment can improve the standard cancer staging criteria and supported the incorporation of nPD-L1 expression detection in CTCs detection in such models.

Circulating tumor cells (CTCs) detach from primary tumors and enter the bloodstream and thus can be the seeds of metastasis. Increasing evidence has proven that the presence of CTCs in the blood of cancer patients parallels their tumor burden and response to therapy^1^,^2^,^3^,^4^,^5^,^6^,^7^. Studies performed in our laboratory as well as by other researchers have indicated that changes in CTC counts are related to therapeutic response. Although CTC count changes are good indicators of response of cancer to drug-based treatments, the need to identify cancer patients at highest risk for aggressive disease is increasing and calls for the identification of reliable protein biomarkers that can be used in conjunction with the CTC count to assess the prognostic significance of these biomarkers.

In a race toward the identification of prognostic biomarkers for different cancers, researchers have discovered several new molecules. One of the most prevalent markers detected is the cell surface glycoprotein programmed death-ligand 1 (PD-L1) (also called B7-H1 and CD274). Authors have reported aberrant expression of PD-L1 in several cancer types^8^,^9^,^10^,^11^ and that this aberrant expression is associated with poor survival of several solid tumors^12^. Interestingly, analysis of PD-L1 expression in CTCs is in the exploratory stages. Mazel *et al.* have recently demonstrated the frequent expression of surface PD-L1 expression on metastatic circulating tumor cells in hormone receptor-positive breast cancer patients^13^.

Although PD-L1 is mainly a membrane protein, Ghebeh *et al.*^14^ reported that treatment with doxorubicin downregulates cell surface PD-L1 expression and upregulates its nuclear expression in breast cancer cells, making them chemoresistant. The aberrant expression of PD-L1 in different types of cancers along with mislocalization of it in the nucleus, which promotes drug resistance, indicating poor prognosis for cancer in patients given chemotherapy, prompted us to detect nuclear PD-L1 (nPD-L1) expression in CTCs in the present study. Because CTCs are believed to withstand harsh environments in the blood apart from exposure to chemotherapy, we sought to determine whether expression of nPD-L1 in CTCs has prognostic significance for colorectal and prostate cancer.

## Materials and Methods

### Study cohort

This retrospective study was approved by The University of Texas MD Anderson Cancer Center Institutional Review Board. Written informed consent to participate in the study was obtained from all patients. Patients of any age with metastatic colorectal cancer refractory to treatment with 5-fluorouracil or with metastatic prostate cancer who were undergoing palliative chemotherapy at MD Anderson were eligible for this study. Routine diagnostic workup included diagnostic imaging, chest X-rays, bone scans, blood sampling, and clinical examination. Peripheral blood samples were collected from 62 metastatic colon cancer patients and 30 metastatic prostate cancer patients for this study. Clinicopathological information was recorded for all patients at the time of blood collection. Response Evaluation Criteria In Solid Tumors guidelines were used to evaluate each patient’s disease status (responding/stable or nonresponding/progression). Because this was a pilot study evaluating the prognostic role of nPD-L1, patients in different cycles of treatment were recruited, and blood was collected at random time points during routine evaluation. Blood samples obtained from five healthy volunteers were tested to determine the specificity of the monoclonal anti-cell-surface vimentin (CSV) 84-1 antibody for cancer cells. Twenty-seven colon cancer and seven prostate cancer patients died over the course of this study. All methods used in this study were performed in accordance with approved guidelines from The University of Texas MD Anderson Cancer Center Institutional Review Board.

### Blood collection and processing

Human blood samples used for CTC analysis were obtained after informed consent was obtained from the patients and healthy donors as per the MD Anderson Institutional Review Board protocol. CTC detection in the samples was conducted as a retrospective study. No attempts to reach a defined statistical power were made. For any given blood draw, a maximum of 7.5 ml of blood was obtained using CPT Vacutainer tubes (BD Biosciences). Single nucleated cells were isolated within 48 h of blood collection as per the manufacturer’s recommendation. Cells were then washed in phosphate-buffered saline (PBS) and used for further analysis. Neither patients nor clinicians were informed of the results of the CTC analysis.

### 84-1^+^ cell selection

84-1^+^ colorectal and prostate cancer cell selection was performed to detect cell-surface vimentin (CSV)^+^ cells. First, CD45^+^ cells were depleted using an EasySep human CD45 depletion kit (STEMCELL Technologies) according to the manufacturer’s recommendation. To minimize nonspecific binding, an antibody against the human Fc receptor (Miltenyi Biotec) was added to the BSA cocktail. Second, the CD45^−^ cell fraction from blood was subjected to 84-1^+^ selection. Briefly, cells were labeled with the 84-1 antibody; mouse IgG-binding microbeads (Miltenyi Biotec) were added to the mixture later. 84-1^+^ cells were then extracted from CD45- cell population using a magnetic column (Miltenyi Biotec) according to the manufacturer’s recommendation. The CTCs thus obtained were 84-1^+^ and CD45^−^ and ready for further analysis. CTCs were then transferred onto glass slides that were fixed using CytoFuge.

### Cell lines and transfection

HCT-116 colorectal cancer and HEK-293 human embryonic kidney cells were obtained from the American Type Culture Collection. Cells were grown in Dulbecco’s modified Eagle’s medium/F12 medium (Sigma-Aldrich) with 10% fetal bovine serum (Gibco, Invitrogen), 1% L-glutamine (Gibco, Invitrogen), and 0.1% penicillin/streptomycin (Gibco, Invitrogen). The cells were maintained at 37 °C in 5% CO_2_. Transfection of PD-L1 in HEK-293 cells was performed using Lipofectamine 3000 (ThermoFisher) as recommended by the manufacturer. Cells after 24 h transfection were used for flow cytometric and confocal analysis.

### Microscopic image capture and analysis

For *in vitro* analysis of cells, 5000 cells per chamber (8 well chamber) were grown on Lab-Tek eight-well Permanox chamber slides (Thermo Fisher Scientific). For intracellular staining of either cells *in vitro* or CTCs, the cells were fixed using 4% paraformaldehyde for 15 min, washed with PBS (pH 7.4), blocked in 10% fetal bovine serum with 0.25% NP-40 for 1 h, and labeled with the 84-1 antibody (1:100) and an anti-PD-L1 antibody (AHP-1703l; AbD Serotec) overnight at 4 °C. Cells were then rinsed in PBS (pH 7.4) and stained with Alexa Fluor 555 or 647 secondary antibodies (1:250; Invitrogen). For nuclear staining, SYTOX green (1:500; Invitrogen) was incorporated into blocking cocktai**l** along with a secondary antibody for 60 min. The cells were then washed with PBS (pH 7.4) three times for 15 min each and mounted using SlowFade Antifade reagent (Invitrogen). For confocal analysis, images of cells were acquired at 8 bits with a Zeiss LSM 510 confocal microscope and the LSM 5 3.2 image capture and analysis software program (Zeiss). A 63x water-immersion objective lens (NA, 1.0) was used with digital zoom for image capture. All images were acquired by the same operator at the same intensity and photodetector gain to allow for quantitative comparisons of relative levels of immunoreactivity between different samples. nPD-L1^+^ cells were scored for nPD-L1 presence or absence based on the nuclear localization of PD-L1 in them. Cells with both nuclear and membrane expression of PD-L1 were included in the nPD-L1^+^ population, whereas cells with only membrane expression of PD-L1 were included in the nPD-L1^−^ population.

### Flow cytometry

Cells (5 × 10^5^) were detached from cell culture dishes using a nonenzymatic dissociation buffer, washed, and fixed in 4% paraformaldehyde for 20 min on ice in the dark. For PD-L1 analysis in the cells, cells were stained with an anti-PD-L1 antibody (1:100); a rabbit primary antibody (Invitrogen) was used as an isotype control. Later, cells were rinsed twice in PBS and labeled for the Alexa Fluor 555 secondary antibody. Cells were then washed twice in PBS and used for data acquisition immediately with an Attune flow cytometer (Applied Biosystems). Fifty thousand cells were counted for the analysis. Data were later analyzed using the FlowJo software program (Tree Star).

### Statistical analysis

All statistical analyses were performed using the Prism software program (GraphPad Software). *P* values less than 0.05 were considered significant. Comparison of survival curves for individual groups of <5 or >5 CTCs and nPDL1^+^ or nPDL1- cells was performed using the log-rank (Mantel-Cox) test. Hazard ratios (HRs) along with 95% confidence intervals (CIs) were represented in the data. An observed HR greater than 1 indicated a worse outcome for the nPD-L1^+^ group than for the nPD-L1^−^ group and was considered statistically significant if the 95% CI did not overlap 1.

## Results

### nPD-L1 expression in cancer cells *in vitro*

**Figure 1:**
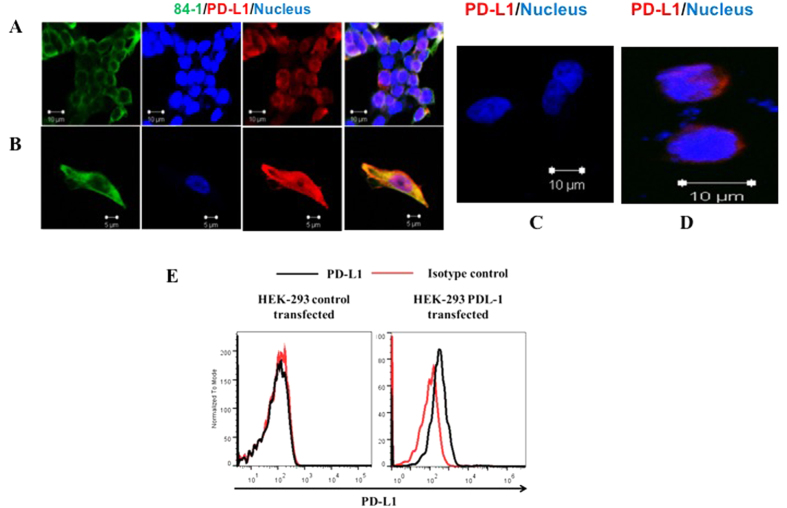
Immunofluorescent staining of HCT-116 and HEK-293 cells for PD-L1 (red), anti-CSV 84-1 (green), and nuclei (blue). (**A**) Stains as a group of cells. (**B**) Stains of single cell. (**C**) HEK-293 cells stained for PD-L1 (red) and nuclei (blue). (**D**) HEK-293 cells transfected with PD-L1 stained for PD-L1 (red) and nuclei (blue). The images were taken using confocal microscopy. Scale bar, 10 μm. (**E**) Flow cytometric evaluation of intracellular PD-L1 expression vector control-transfected (left panel) and PD-L1–transfected (right panel) HEK-293 cells.

We tested for nPD-L1 expression in CTC analysis because we made an interesting observation in our initial study of HCT-116 cells *in vitro.* In short, HCT-116 cells exhibited both cytoplasmic/membranous and nuclear localization of PD-L1. The rate of nuclear localization remained below 10% when we densely plated the cells (Fig. 1A), whereas the majority of the cells exhibited membranous and cytoplasmic PD-L1 expression. However, when we plated the cells individually (Fig. 1B), the extent of nuclear localization of PD-L1 was about 90%, suggesting association of the nPD-L1 phenotype with cells lacking cell-cell contact, which is the case for human CTCs.

To determine whether the detected nPD-L1 was true PD-L1, we validated the specificity of the PD-L1-detecting anti-PD-L1 antibody. As a control for the antibody detection, we transfected HEK-293 cells not expressing PD-L1 with a plasmid encoding PD-L1. Our results indicated that the anti-PD-L1 antibody we used was very specific for PD-L1 because it was unable to detect PD-L1 expression in the HEK-293 cells (Fig. 1C), whereas we detected PD-L1 expression in PD-L1 transfected cells (Fig. 1D). However, the rate of nPD-L1 expression in HEK-293 cells was lower than that observed in cancer cells, possibly because of an absence of machinery that translocates PD-L1 to the nucleus.

Also, we tested HEK-293 cells (control- and PD-L1–transfected) for detection of PD-L1 expression using flow cytometry. Our results demonstrated that PD-L1 expression was initially undetectable in these cells but was detectable after transfection (Fig. 1E) as observed from the shift in the histogram.

### nPD-L1 detection in human CTCs

**Figure 2:**
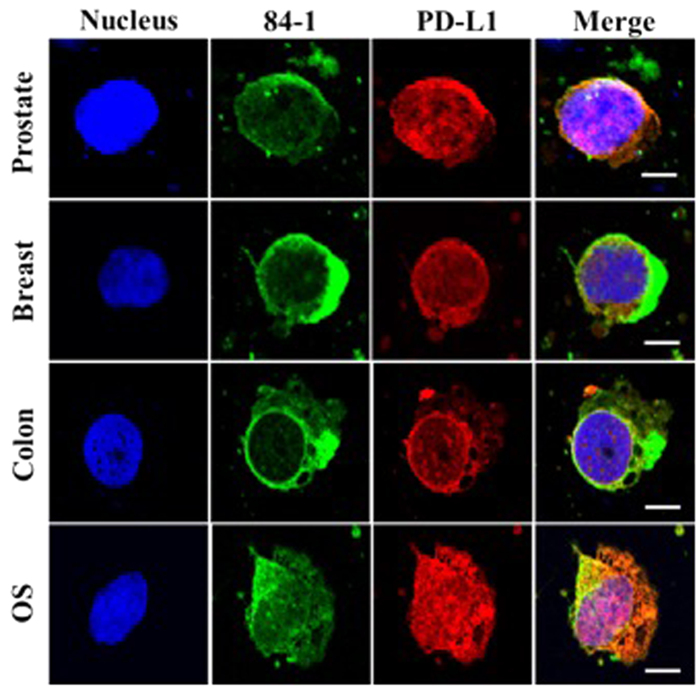
PD-L1 expression in CTCs isolated from prostate cancer, breast cancer, colon cancer, and osteosarcoma patients. Cells were stained for CSV (green) using the 84-1 antibody, PD-L1 (red), and SYTOX green (pseudo blue). The images were taken using confocal microscopy. Scale bar, 10 μm.

Given that detection of nPD-L1 is more prominent in cells lacking cell-cell contacts in an *in vitro* setup, we hypothesized that cancer cells that detach from a tumor and enter the bloodstream (CTCs) exhibit nPD-L1 localization. We previously showed that the 84-1 method that isolates CTCs in patients with epithelial and mesenchymal cancers. Therefore, we tested blood samples collected from prostate, breast, and colorectal cancer and osteosarcoma patients for CTC enumeration using this method^1^,^2^,^15^. Our preliminary analysis for PD-L1 detection in CTCs using confocal microscopy suggested that expression of PD-L1 is heterogeneous, with detectable expression in the cytoplasm, membrane, and nucleus. nPD-L1 expression was prominent in CTCs isolated from different tumor types (Fig. 2). To the best of our knowledge, this is the first report of nuclear localization of PD-L1 in CTCs. Blood samples obtained from healthy donors were negative for CTCs.

### Correlation between nPDL1 expression and survival in colorectal cancer patients

**Figure 3:**
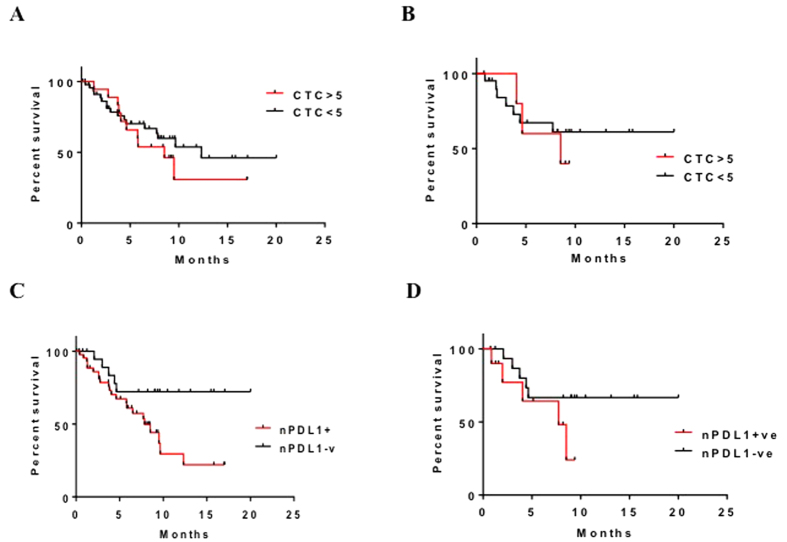
Overall Survival (OS) and Progression Free Survival determination in colorectal cancer patients. (**A**) OS durations in CTC^+^ patients with colon cancer. (**B**) PFS durations in CTC^+^ patients with colon cancer. (**C**) OS durations in nPDL-1^+^/CTC^+^ patients with colon cancer. (**D**) PFS durations in nPDL-1^+^/CTC^+^ patients with colon cancer.

Given that nPD-L1 expression is detectable in human CTCs, we sought to determine whether detection of it has prognostic relevance for cancer. We collected CTCs from colorectal cancer patients and analyzed them for nPD-L1 expression. To assess the prognostic impact of nPD-L1 expression, we counted the cells with and without nPD-L1 expression. We considered a patient to be nPD-L1^+^ if more than 50% of his or her CTCs expressed nPD-L1 (Supplementary Table S1). We also evaluated the prognostic significance of total CTC counts (<5 versus >5 CTCs per 7.5 mL of blood) and nPDL1 expression (positive versus negative). Total CTC counts yielded an HR for overall survival (OS) of 1.353 (95% CI, 0.5939–3.0820; *n* = 67; *P* = 0.4718) (Fig. 3A) and an HR for progression-free survival (PFS) of 1.373 (95% CI, 0.3221–5.8480; *n* = 27; *P* = 0.6685) (Fig. 3B). Analysis of nPD-L1 expressing CTCs yielded an HR for OS of 2.437 (95% CI, 1.110–5.350; *n* = 67; *P* = 0.0264) (Fig. 3C) and for PFS of 2.599 (95% CI, 0.6681–10.1100; *n* = 27; *P* = 0.1682) (Fig. 3D). These results indicated that nPD-L1 expression in CTCs was associated with a markedly worse outcome in terms of OS than was a lack of this expression but that its expression was not associated with significantly worse PFS in colorectal cancer patients.

### Correlation between nPDL1 expression and survival in prostate cancer patients

**Figure 4:**
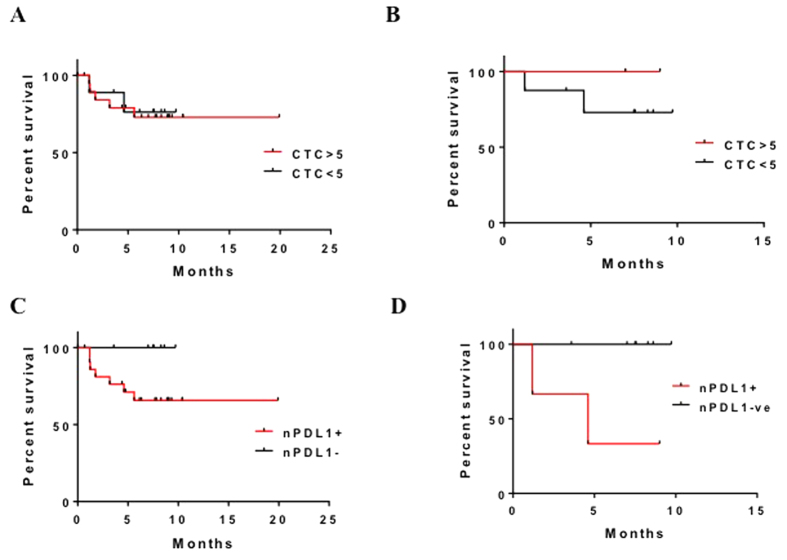
Overall Survival (OS) and Progression Free Survival determination in prostate cancer patients. (**A**) OS durations in CTC^+^ patients with prostate cancer. (**B**) PFS durations in CTC^+^ patients with prostate cancer. (**C**) OS durations in nPDL-1^+^/CTC^+^ patients with prostate cancer. (**D**) PFS durations in nPDL-1^+^/CTC^+^ patients with prostate cancer.

We also analyzed prognostic data on a small group of prostate cancer patients for whom epithelial-mesenchymal transitioned (EMT) CTCs were enumerated and assessed for their prognostic significance (Supplementary Table S2). Total CTC counts yielded an HR for OS of 1.182 (95% CI, 0.2400–5.8230; *n* = 30; *P* = 0.8369) (Fig. 4A) and for PFS of 0.2739 (95% CI, 0.009854–7.614000; *n* = 10; *P* = 0.4452) (Fig. 4B). nPD-L1 CTC counts yielded an HR for OS of 4.060 (95% CI, 0.7684–21.4600; *n* = 30; *P* = 0.0990) (Fig. 4C) and for PFS of 38.39 (95% CI, 1.714–859.700; *n* = 10; *P* = 0.0215) (Fig. 4D). The results indicated that nPD-L1 expression in CTCs was associated with markedly worse PFS but not OS. Given the small number of patients analyzed in this group, this association must be analyzed in a larger cohort to better determine impact of nPD-L1 localization as a prognostic marker.

## Discussion

Patients with colorectal cancer receive adjuvant chemotherapy after undergoing curative surgery to prevent recurrence or metastasis. Nevertheless, only a few subsets of patients benefit from such treatment. Although advancements in the field of cancer diagnostics have provided new possibilities for prognosis, new prognostic markers that can identify cancer patients who have not benefited from treatment and that have prognostic relevance for predicting survival are needed. CTCs have recently gained momentum as probes that guide monitoring of therapeutic response in cancer patients^16^,^17^,^18^. For example, EMT cells are recognized for their drug resistance, stemness, and ability to invade surrounding areas^19^,^20^,^21^. Keeping these characteristics of EMT cells and CTCs in mind, EMT CTCs may be key determinants of survival in patients undergoing chemotherapy and would be helpful in understanding prognosis for cancer. We and other researchers have reported on the detection of EMT CTCs in breast, colorectal cancers and their association with aggressive cancer phenotypes^1^,^2^,^17^. However, their role as prognostic indicators for cancer remains undetermined.

We aimed to evaluate the role of EMT CTCs isolated from the peripheral blood of cancer patients using CSV as a marker to identify their prognostic relevance. However, CTC counts of 1, 2, 3, 4, and greater than 5 were not associated with significantly worse outcomes in terms of PFS and OS. Among the main factors that may have influenced this outcome were the need for a longer follow-up duration and uniformity in CTC determination at different time points. Of note is that the majority of the studies in which CTCs were enumerated using the CellSearch test used patients who did not undergo treatment; these CTC counts were associated with poor survival^22^,^23^,^24^,^25^. However, in patients undergoing curative treatment, these counts were not associated with poor survival, suggesting that the cells detected using CellSearch either underwent EMT or were nearly undetectable. These limitations prompted us to look for new markers of EMT, the expression of which is altered by therapy.

Blocking interaction between the programmed cell death-1 protein and its ligand PD-L1 is reported to produce extraordinary antitumor responses, and researchers in a number of clinical trials are assessing the prognostic relevance of PD-L1 in cancers. Although investigators have performed a large number of CTC studies, there has been only one study that has detected the expression of PD-L1 in CTCs isolated from blood of cancer patients^13^, however this study doesn’t provide compartmentalization of PD-L1 status in CTCs. To fill this gap in knowledge about CTCs, we evaluated PD-L1 expression in different EMT CTCs isolated from cancer patients using the 84-1 method developed in our laboratory. Our initial goal was to evaluate PD-L1 expression in the membranes and cytoplasm of CTCs. However, we were surprised to observe PD-L1 expression in the nuclei of the majority of CTCs, indicating the possibility of mistranslocation of PD-L1 in these cells. A thorough literature search shed light on nPD-L1 expression in breast cancer patients and its possible role in breast cancer progression. These data provided the impetus for us to analyze nPD-L1 expression in patients with different cancer.

To confirm nPD-L1 localization in CTCs, we tested colorectal cancer cells cultured *in vitro* to see if they expressed nPD-L1. Our results indicated lower levels of nPD-L1 expression when the cells were in a larger cluster of cells. However, when we individually plated the cells and analyzed them for PD-L1 expression, we found that they tended to exhibit more nuclear localization than in a cluster of cells. These results prompted us to ask if all types of cancers have this phenomenon. To answer that question, we examined CTCs obtained from breast cancer, prostate cancer, and osteosarcoma patients for nPD-L1 expression. Our results demonstrated that these types of cancer cells do have nPD-L1 expression. A possible explanation for this is that when cells are examined individually, they tend to have patterns of signaling activation different from that in cells examined in groups that promote nuclear translocation of PD-L1. Ghebeh *et al.*^14^ reported that chemotherapy induces nuclear translocation of PD-L1 and suggested that PD-L1 has functions other than inhibition of T cells. Also, authors reported expression of B7-H3, a member of the B7 superfamily, in the nuclei of colon cancer cells and that this expression was strongly correlated with poor outcome^26^. These findings provide further evidence that nuclear translocation of PD-L1 has great prognostic value for cancer.

We reported that detection of CTCs from blood of cancer patients using the 84-1 method predicted therapeutic response in cancer patients. Because PD-L1 expression (both membranous and cytoplasmic) in CTCs did not have prognostic significance, we focused the present study on evaluating nPD-L1 expression in CTCs and its prognostic relevance for cancer. Our results indicated that nPD-L1 expression predicts poor prognosis for colorectal cancer with respect to OS and for prostate cancer with respect to PFS. These results indicated that nuclear localization of PD-L1 may be involved in cancer progression and metastasis and suggested that nuclear PD-L1 expression in CTCs may be a useful prognostic marker for different cancers. However, these results must be confirmed in multicenter prospective studies.

## Additional Information

**How to cite this article**: Satelli, A. *et al.* Potential role of nuclear PD-L1 expression in cell-surface vimentin positive circulating tumor cells as a prognostic marker in cancer patients. *Sci. Rep.* **6**, 28910; doi: 10.1038/srep28910 (2016).

## Supplementary Material

Supplementary Information

## References

[b1] Satelli A. *et al.* Epithelial-mesenchymal transitioned circulating tumor cells capture for detecting tumor progression. Clin Cancer Res 21, 899–906 (2015). 25516888 10.1158/1078-0432.CCR-14-0894 PMC4334736

[b2] Satelli A. *et al.* Universal marker and detection tool for human sarcoma circulating tumor cells. Cancer Res 74, 1645–1650 (2014). 24448245 10.1158/0008-5472.CAN-13-1739 PMC3959622

[b3] Pantel K. & Alix-Panabieres C. The potential of circulating tumor cells as a liquid biopsy to guide therapy in prostate cancer. Cancer Discov 2, 974–975 (2012). 23093252 10.1158/2159-8290.CD-12-0432

[b4] Lowes L. E. *et al.* The significance of circulating tumor cells in prostate cancer patients undergoing adjuvant or salvage radiation therapy. Prostate Cancer Prostatic Dis 18(4), 358–64 (2015). 26238233 10.1038/pcan.2015.36 PMC4788488

[b5] Ren C. *et al.* Circulating tumor cells in breast cancer beyond the genotype of primary tumor for tailored therapy. Int J Cancer 138(7), 1586–600 (2016). 26178386 10.1002/ijc.29679

[b6] Agelaki S. *et al.* Efficacy of Lapatinib in Therapy-Resistant HER2-Positive Circulating Tumor Cells in Metastatic Breast Cancer. PLoS One 10, e0123683 (2015). 26083256 10.1371/journal.pone.0123683 PMC4471111

[b7] Dorsey J. F. *et al.* Tracking viable circulating tumor cells (CTCs) in the peripheral blood of non-small cell lung cancer (NSCLC) patients undergoing definitive radiation therapy: pilot study results. Cancer 121, 139–149 (2015). 25241991 10.1002/cncr.28975 PMC4270850

[b8] Bertucci F. *et al.* PDL1 expression is an independent prognostic factor in localized GIST. Oncoimmunology 4, e1002729 (2015). 26155391 10.1080/2162402X.2014.1002729 PMC4485716

[b9] Bertucci F. *et al.* PDL1 expression in inflammatory breast cancer is frequent and predicts for the pathological response to chemotherapy. Oncotarget 6, 13506–13519 (2015). 25940795 10.18632/oncotarget.3642 PMC4537030

[b10] Sabatier R. *et al.* Prognostic and predictive value of PDL1 expression in breast cancer. Oncotarget 6, 5449–5464 (2015). 25669979 10.18632/oncotarget.3216 PMC4467160

[b11] Hasan A., Ghebeh H., Lehe C., Ahmad R. & Dermime S. Therapeutic targeting of B7-H1 in breast cancer. Expert Opin Ther Targets 15, 1211–1225 (2011). 21870995 10.1517/14728222.2011.613826

[b12] Wu P., Wu D., Li L., Chai Y. & Huang J. PD-L1 and Survival in Solid Tumors: A Meta-Analysis. PLoS One 10, e0131403 (2015). 26114883 10.1371/journal.pone.0131403 PMC4483169

[b13] Mazel M. *et al.* Frequent expression of PD-L1 on circulating breast cancer cells. Mol Oncol 9, 1773–1782 (2015). 26093818 10.1016/j.molonc.2015.05.009 PMC5528721

[b14] Ghebeh H. *et al.* Doxorubicin downregulates cell surface B7-H1 expression and upregulates its nuclear expression in breast cancer cells: role of B7-H1 as an anti-apoptotic molecule. Breast Cancer Res 12, R48 (2010). 20626886 10.1186/bcr2605 PMC2949635

[b15] Satelli A., Brownlee Z., Mitra A., Meng Q. H. & Li S. Circulating tumor cell enumeration with a combination of epithelial cell adhesion molecule- and cell-surface vimentin-based methods for monitoring breast cancer therapeutic response. Clin Chem 61, 259–266 (2015). 25336717 10.1373/clinchem.2014.228122 PMC4360893

[b16] Pantel K. & Speicher M. R. The biology of circulating tumor cells. Oncogene, doi: 10.1038/onc (2015). 26050619

[b17] McInnes L. M. *et al.* Clinical implications of circulating tumor cells of breast cancer patients: role of epithelial-mesenchymal plasticity. Front Oncol, doi: 10.3389 (2015). 10.3389/fonc.2015.00042 PMC4341429 25767772

[b18] Liu H. *et al.* The biological and clinical importance of epithelial-mesenchymal transition in circulating tumor cells. J Cancer Res Clin Oncol 141, 189–201 (2015). 24965746 10.1007/s00432-014-1752-x PMC11823907

[b19] Mitra A., Mishra L. & Li S. EMT, CTCs and CSCs in tumor relapse and drug-resistance. Oncotarget 6, 10697–10711 (2015). 25986923 10.18632/oncotarget.4037 PMC4484413

[b20] Bill R. & Christofori G. The relevance of EMT in breast cancer metastasis: Correlation or causality? FEBS Lett 589, 1577–1587 (2015). 25979173 10.1016/j.febslet.2015.05.002

[b21] Zoni E., van der Pluijm G., Gray P. C. & Kruithof-de Julio M. Epithelial Plasticity in Cancer: Unmasking a MicroRNA Network for TGF-beta-, Notch-, and Wnt-Mediated EMT. J Oncol 198967 (2015). 25883651 10.1155/2015/198967 PMC4390187

[b22] Beije N., Jager A. & Sleijfer S. Circulating tumor cell enumeration by the CellSearch system: the clinician’s guide to breast cancer treatment? Cancer Treat Rev 41, 144–150 (2015). 25542852 10.1016/j.ctrv.2014.12.008

[b23] Adams D. L. *et al.* Cytometric characterization of circulating tumor cells captured by microfiltration and their correlation to the cellsearch((R)) CTC test. Cytometry A 87, 137–144 (2015). 25515318 10.1002/cyto.a.22613

[b24] Truini A. *et al.* Clinical Applications of Circulating Tumor Cells in Lung Cancer Patients by CellSearch System. Front Oncol 4, 242 (2015). 25237652 10.3389/fonc.2014.00242 PMC4154446

[b25] Raimondi C., Gradilone A., Naso G., Cortesi E. & Gazzaniga P. Clinical utility of circulating tumor cell counting through CellSearch((R)): the dilemma of a concept suspended in Limbo. Onco Targets Ther 7, 619–625 (2014). 24790460 10.2147/OTT.S46200 PMC4000244

[b26] Ingebrigtsen V. A. *et al.* B7-H3 expression in colorectal cancer: nuclear localization strongly predicts poor outcome in colon cancer. Int J Cancer 131, 2528–2536 (2012). 22473715 10.1002/ijc.27566

