# Supplementary material for: Potential role of nuclear PD-L1 expression in cell-surface vimentin positive circulating tumor cells as a prognostic marker in cancer patients
Source: Sci Rep. 2016 Jul 1;6:28910. doi: 10.1038/srep28910 (PMC4929464; doi:10.1038/srep28910)
Supplement: Supplementary Information [file srep28910-s1.pdf]

Potential role of nuclear PD-L1 expression in cell-surface vimentin positive circulating tumor cells as a prognostic marker in cancer patients

Arun Satelli[1], Izhar Singh Batth[1], Zachary Brownlee[1], Christina Rojas[1], Qing H. Meng[2], Scott Kopetz[3] & Shulin Li[1,4,*]

[1]Department of Pediatrics, The University of Texas MD Anderson Cancer Center, Houston, Texas. [2]Department of Laboratory Medicine, The University of Texas MD Anderson Cancer Center, Houston, Texas. [3]Departments of Surgical Oncology and Molecular and Cellular Oncology, The University of Texas MD Anderson Cancer Center, Houston, Texas. [4]The University of Texas Graduate School of Biomedical Sciences, Houston, Texas. *Correspondence and requests for materials should be addressed to S.L. (sli4@mdanderson.org)

Short Title: Nuclear PDL-1 in circulating tumor cells

Keywords: PD-L1, Vimentin, CTC, Colon cancer, Prostate cancer

Supplementary Table S1. Survival durations, CTC counts, and nPD-L1 positivity in colon cancer patients.

Patient number	Survival duration (months)	CTC count	≥50% positive for nPD-L1
1	3.780821918	5	No
2	13.11780822	1	No
3	11.80273973	2	No
5	9.60000000	5	No
6	8.219178082	1	No
8	4.635616438	7	No
9	15.25479452	4	No
10	10.48767123	4	No
11	2.860273973	4	No
12	0.098630137	4	No
13	4.405479452	4	No
14	6.509589041	4	No
15	3.912328767	4	Yes
17	9.369863014	3	No
18	2.991780822	5	No
19	8.186301370	14	No
21	13.57808219	4	Yes
22	2.82739726	2	Yes
23	6.969863014	2	Yes
25	1.380821918	6	Yes
26	1.578082192	2	Yes
27	7.989041096	4	Yes
28	2.334246575	20	Yes
29	5.983561644	5	Yes
30	2.695890411	3	Yes
31	4.438356164	3	Yes
32	3.715068493	4	Yes
33	0.854794521	17	Yes
35	9.205479452	6	Yes
36	1.84109589	1	Yes
37	9.665753425	1	Yes
38	1.282191781	1	Yes
39	1.052054795	2	Yes
40	4.043835616	3	Yes
41	1.41369863	2	Yes
42	13.38082192	5	Yes
43	0.756164384	1	Yes
44	3.879452055	6	Yes
45	15.81369863	3	Yes
46	5.128767123	1	Yes
47	0.854794521	2	Yes
48	8.18630137	1	Yes
49	0.887671233	2	Yes
50	2.367123288	3	Yes
51	1.117808219	11	Yes
52	9.501369863	12	Yes
53	3.846575342	5	Yes
54	0.98630137	3	Yes
55	6.082191781	2	Yes
56	2.761643836	1	Yes
58	4.767123288	8	Yes
59	16.99726027	10	Yes
60	0.756164384	3	No
61	15.81369863	1	Yes
62	20.02191781	2	No
63	15.48493151	2	No
64	9.073972603	1	No
65	12.32876712	2	Yes
66	4.701369863	2	Yes
67	17.03013699	12	Yes
70	1.249315068	9	No

Supplementary Table S2. Survival durations, CTC counts, and nPD-L1 positivity in prostate cancer patients.

Patient number	Survival duration (months)	CTC count	≥50% positive for nPDL1
P4	8.284931507	35	Yes
P10	4.602739726	1	Yes
P11	3.189041096	134	Yes
P12	9.336986301	91	Yes
P13	10.38904110	5	Yes
P14	4.767123288	14	Yes
P15	9.731506849	4	No
P16	10.42191781	22	Yes
P18	7.824657534	890	Yes
P19	0.032876712	23	Yes
P20	9.008219178	93	Yes
P21	8.876712329	76	Yes
P22	9.073972603	42	Yes
P23	1.249315068	10	Yes
P24	5.621917808	21	Yes
P27	0.690410959	35	Yes
P28	1.183561644	56	Yes
P35	8.613698630	1	No
P37	7.002739726	18	No
P38	4.405479452	9	Yes
P41	3.583561644	1	No
P42	8.284931507	1	No
P45	7.693150685	5	Yes
P46	7.561643836	1	No
P47	1.183561644	3	Yes
P51	7.495890411	3	No
P52	6.345205479	6	Yes
P54	6.147945205	1	Yes
P56	19.92328767	6	Yes
P57	1.775342466	6	Yes
